# Supplementary material for: The genome sequence of Nathusius’ pipistrelle, Pipistrellus nathusii (Keyserling & Blasius, 1839) (Chiroptera: Vespertilionidae)
Source: Wellcome Open Res. 2026 Apr 22;11:234. [Version 1] doi: 10.12688/wellcomeopenres.26312.1 (PMC13270188; doi:10.12688/wellcomeopenres.26312.1)
Supplement: Peer review history

# Reviewer report: Reviewer response for version 1

**DOI:** 10.21956/wellcomeopenres.28977.r157928

**Published:** 2026-06-15

**Authors:** Xiuguang Mao

**Affiliations:** 1 East China Normal University, Shanghai, China

This manuscript provides a high-quality chromosome-level genome assembly for *Pipistrellus nathusii*. Together with the previous high-quality assembly of this species, these genome resources will greatly contribute to bat research. For the current version of the manuscript, I have only three minor comments:

 1) "The mitochondrial genome was assembled using MitoHiFi." How much PacBio HiFi data (all or a subset — please be specific) was used for the mitochondrial genome assembly?

 2) "The final assembly has a total length of 1,961.71 Mb," which is larger than the haploid genome size estimated by GenomeScope 2.0 analysis (1,767.67 Mb). What is the reason for this inconsistency?

 3) In Table 3, if the chromosomes are numbered based on their length, why is Chr8 (89.97 Mb) longer than Chr7 (86.80 Mb)?

Are sufficient details of methods and materials provided to allow replication by others?

Yes

Is the rationale for creating the dataset(s) clearly described?

Yes

Are the datasets clearly presented in a useable and accessible format?

Yes

Are the protocols appropriate and is the work technically sound?

Yes

Reviewer Expertise:

Comparative genomics, genome assembly, evolutionary biology, speciation, bats

I confirm that I have read this submission and believe that I have an appropriate level of expertise to confirm that it is of an acceptable scientific standard.

# Reviewer report: Reviewer response for version 1

**DOI:** 10.21956/wellcomeopenres.28977.r154089

**Published:** 2026-05-12

**Authors:** Luciano Chaves Franco Filho

**Affiliations:** 1 Instituto Evandro Chagas, Ananindeua, Brazil

The manuscript presents a chromosome-level genome assembly of *Pipistrellus nathusii* (Nathusius’ pipistrelle), generated using PacBio HiFi, Hi-C, and RNA-seq technologies within the Bat1K and Darwin Tree of Life initiatives. The manuscript is well organized and presents relevant genomic data for *Pipistrellus nathusii*. The methodologies and assembly metrics are adequately described, and the study provides a useful genomic resource for the scientific community. I consider the manuscript suitable for indexing following standard editorial evaluation.

Are sufficient details of methods and materials provided to allow replication by others?

Yes

Is the rationale for creating the dataset(s) clearly described?

Yes

Are the datasets clearly presented in a useable and accessible format?

Yes

Are the protocols appropriate and is the work technically sound?

Yes

Reviewer Expertise:

I have expertise in genomics, metagenomics, molecular biology, and molecular diagnostics applied to infectious diseases and public health. My work focuses on genomic surveillance, pathogen detection, sequencing technologies, and the application of molecular approaches to investigate microbial diversity, antimicrobial resistance, and epidemiological monitoring.

I confirm that I have read this submission and believe that I have an appropriate level of expertise to confirm that it is of an acceptable scientific standard.
